# One-Step Affinity Purification of Leucine-Rich α_2_-Glycoproteins from Snake Sera and Characterization of Their Phospholipase A_2_-Inhibitory Activities as β-Type Phospholipase A_2_ Inhibitors

**DOI:** 10.3390/toxins16030126

**Published:** 2024-03-01

**Authors:** Ryoichi Shirai, Kana Shibata, Shinobu Fujii, Rikiro Fukunaga, Seiji Inoue

**Affiliations:** 1 Department of Biochemistry, Faculty of Pharmacy, Osaka Medical and Pharmaceutical University, Takatsuki 569-1094, Osaka, Japan; ryoichi_shirai@hosp.misasa.tottori.jp (R.S.); shinobu.fujii@ompu.ac.jp (S.F.); rikiro.fukunaga@ompu.ac.jp (R.F.); 2 Misasa Onsen Hospital, Misasa 682-1097, Tottori, Japan; 3 Center for the Advancement of Pharmaceutical Education, Faculty of Pharmacy, Osaka Medical and Pharmaceutical University, Takatsuki 569-1094, Osaka, Japan

**Keywords:** phospholipase A_2_ inhibitor, phospholipase A_2_, leucine-rich α_2_-glycoprotein, cytochrome *c*, Viperidae, Elapidae, Colubridae

## Abstract

Snakes contain three types of phospholipase A_2_ (PLA_2_)-inhibitory proteins in their blood, PLIα, β, and γ, which protect them from their own venom, PLA_2_. PLIβ is the snake ortholog of leucine-rich α_2_ glycoprotein (LRG). Since autologous cytochrome *c* (Cyt *c*) serves as an endogenous ligand for LRG, in this study, we purified snake LRGs from various snake serum samples using Cyt *c* affinity chromatography. All purified snake LRGs were found to be dimers linked by disulfide bonds. *Laticauda semifasciata* and *Naja kaouthia* LRGs showed no inhibitory activity against *L. semifasciata* PLA_2_ and weak inhibitory activity against *Gloydius brevicauda* basic PLA_2_. *Elaphe climacophora* PLIβ had weaker inhibitory activity against *G. brevicauda* basic PLA_2_ than *G. brevicauda* and *Elaphe quadrivirgata* PLIs, which are abundant in blood and known to neutralize *G. brevicauda* basic PLA_2_. *Protobothrops flavoviridis* LRG showed no inhibitory activity against basic venom PLA_2_, PL-X, or *G. brevicauda* basic PLA_2_. Binding analysis of *P. flavoviridis* LRG using surface plasmon resonance showed very strong binding to snake Cyt *c*, followed by that to horse Cyt *c*, weak binding to yeast Cyt *c*, and no binding to *P. flavoviridis* PL-X or BPI/II. We also deduced the amino acid sequences of *L. semifasciata* and *P. flavoviridis* LRG by means of cDNA sequencing and compared them with those of other known sequences of PLIs and LRGs. This study concluded that snake LRG can potentially inhibit basic PLA_2_, but, whether it actually functions as a PLA_2_-inhibitory protein, PLIβ, depends on the snake.

## 1. Introduction

Snakes possess self-protective mechanisms against venom. Three distinct phospholipase A_2_ (PLA_2_)-inhibitory proteins, named PLIα, PLIβ, and PLIγ, have been purified from the serum of the Chinese mamushi, *Gloydius brevicauda* (renamed *Agkistrodon blomhoffii siniticus* according to the present taxonomy) [1]. To date, PLIα and PLIγ have been reported in many snakes [2,3,4]. These inhibitory proteins directly bind to venom PLA_2_s and inhibit their enzymatic activity. PLIαs are generally found in the sera of viperid snakes, and their structure is characterized by the 20 kDa glycosylated subunit with a C-type lectin-like domain [2,5]. They specifically inhibit group II acidic PLA_2_s [6] or basic myotoxic PLA_2_s [7]. PLIγs can inhibit a broad spectrum of group I, II, and III PLA_2_s [6,8,9], and their primary structures contain two tandem repeats of a cysteine pattern characteristic of the three-finger motifs found in Ly-6-related proteins [10]. PLIγ have been identified in the sera of many venomous snakes, including elapid and viperid snakes, as well as non-venomous snakes [2]. PLIβ has nine tandem leucine-rich repeats in its sequence and 33% sequence homology with human leucine-rich α_2_-glycoprotein (LRG) [11]. Human LRG is one of the serum proteins of unknown function and has recently accumulated evidence for its contribution to a wide range of human diseases [12]. Snake PLIβ selectively inhibits group II basic PLA_2_s and is thought to function as part of a self-defense mechanism against its own venom [1]. It was purified from the sera of the viperid snake, *G. brevicauda* [1] and two non-venomous snakes, *Elaphe quadrivirgata* [13] and *Elaphe climacophora* [14], using multi-step purification including ion exchange, gel filtration, and hydrophobic column chromatography. PLIβ transcripts have been found in cDNA libraries from the venom glands and livers of the viperid snakes *Lachesis muta* [15], *Bothrops alternatus*, *Bothrops jararaca*, *Bothrops moojeni*, *Bothrops neuwiedi*, and *Crotalus durissus terrificus* [16]. However, PLIβ has not been reported in an elapid snake. Autologous cytochrome *c* (Cyt *c*) is the endogenous ligand of LRG and PLIβ [17], and PLIβ can be regarded as the snake LRG with PLA_2_-inhibitory activity. Owing to this, LRGs can be easily purified from serum using a Cyt *c* affinity column [17,18].

In the present study, we purified snake LRGs from various snake sera using a Cyt *c* affinity column and investigated whether snake LRGs generally have PLA_2_-inhibitory activity and function as a defense mechanism against snake venom PLA_2_.

## 2. Results

### 2.1. Purification of LRGs from Various Snake Sera

**Figure 1:**
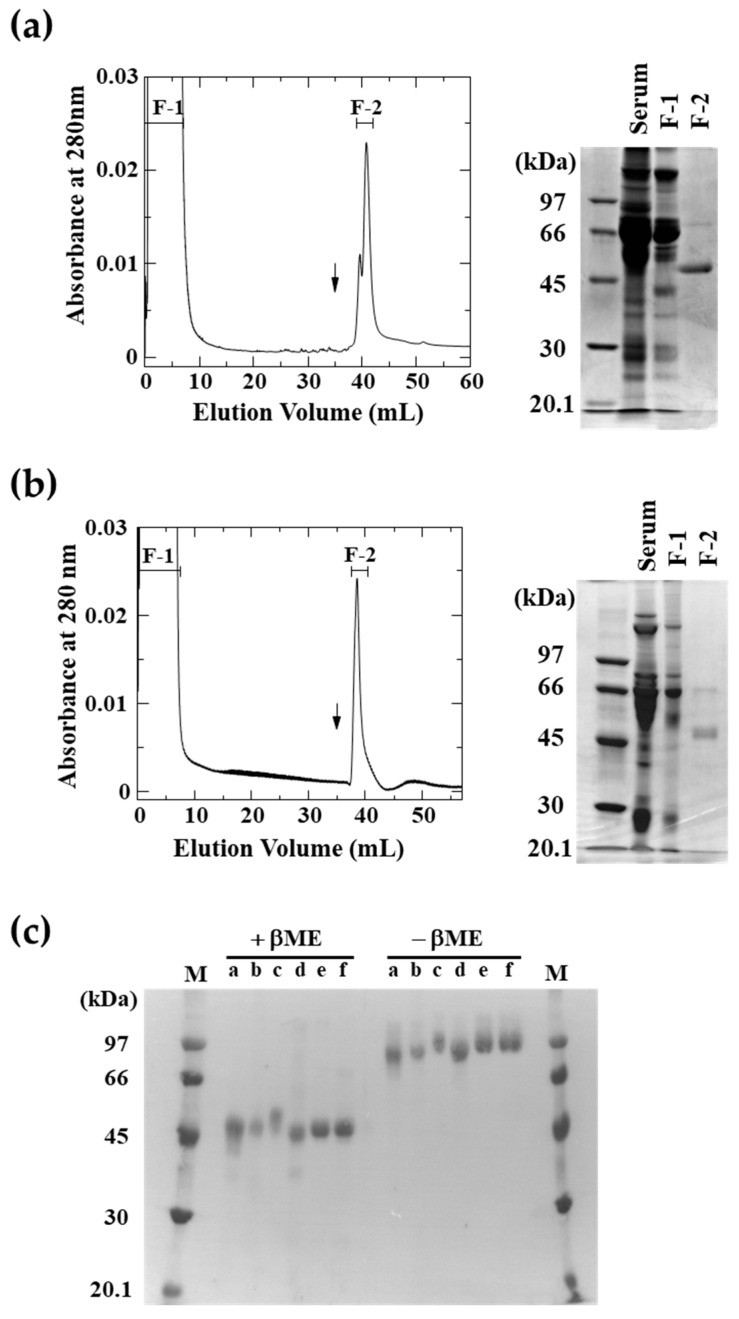
Purification of snake LRGs by means of Cyt *c* affinity chromatography. *Laticauda semifasciata* (**a**) or *Protobothrops flavoviridis* (**b**) serum was applied to a Cyt *c*-coupled HiTrap NHS-activated HP column equilibrated with 20 mM phosphate buffer (pH 7.4) containing 0.15 M NaCl. The column was washed with the same buffer, and the adsorbed proteins were eluted with 100 mM acetate buffer (pH 4.0) containing 0.5 M NaCl (*arrow*). F-1, flow-through fraction; F-2, adsorbed fraction. The whole serum and fractions (F-I and F-II) obtained after Cyt *c* affinity column chromatography were subjected to SDS-PAGE. (**c**) SDS-PAGE of the purified snake LRGs. a, *Gloydius brevicauda* LRG; b, *P. flavoviridis* LRG; c, *Elaphe quadrivirgata* PLIβ purified in a previous study [13]; d, *Elaphe climacophora* LRG; e, *Naja kaouthia* LRG; and f, *L. semifasciata* LRG. LRG, leucine-rich α_2_-glycoprotein; SDS-PAGE, sodium dodecyl sulfate-polyacrylamide gel electrophoresis; βME, β-mercaptoethanol.

Snake PLIβ and human LRG are homologous serum glycoproteins with Cyt *c*-binding activity, indicating that PLIβ is the snake ortholog of LRG [17]. In fact, the PLIβ cDNA sequence was blasted against the blue-ringed sea krait genome using the Ensemble Genome Browser and its synteny was found to be almost identical to that of the human LRG gene, indicating that the PLIβ gene is the same gene as the LRG gene in mammals. Because it has been reported that LRG can be easily purified using Cyt *c* affinity columns [17,18], we attempted to purify snake LRGs from various snake sera using the same technique. LRGs from the sera of *Laticauda semifasciata* and *Protobothrops flavoviridis* that were specifically adsorbed on a Cyt *c* affinity column and eluted with 0.1 M acetate buffer (pH 4.0) containing 0.5 M NaCl showed almost a single 50 kDa band on the sodium dodecyl sulfate-polyacrylamide gel electrophoresis (SDS-PAGE) (Figure 1a,b). Similarly, we also purified snake LRGs from the sera of *G. brevicauda*, *E. climacophora*, and *Naja kaouthia*. The molecular weight of the purified snake LRGs was approximately 50 kDa in the SDS-PAGE in the presence of β-mercaptoethanol, but around 100 kDa in its absence, suggesting that these are dimers linked by disulfide bonds (Figure 1c). Since no monomeric bands are detected under non-reducing conditions, this is probably not an artificial dimerization by purification. The N-terminal 10-amino acid sequences of the purified *G. brevicauda* and *E. climacophora* LRGs were consistent with the previously determined PLIβ sequences [11,14]. The N-terminal amino acid sequences of *L. semifasciata*, *N. kaouthia*, and *P. flavoviridis* LRGs were determined to be Val-Leu-Tyr-X-Pro-Pro-Asp-Pro-Ala, Val-Leu-Tyr-X-Pro-Pro-X-Pro-Ala-Pro-Glu, and Val-Leu-Tyr-X-Pro-Pro-Thr-Pro-Ala-Pro-Glu-Ser-Val-Thr-Glu-Phe-Val-X-Asn-Ser, respectively. The use of Cyt *c* affinity columns makes it possible to purify PLIs from serum in a single step. Table 1 shows the amount of snake LRG recovered from sera using the Cyt *c* affinity column. The *G. brevicauda* serum contained large amounts of LRG (PLIβ), as compared to the other snake sera.

### 2.2. PLA_2_-Inhibitory Activities of the Snake LRGs

**Figure 2:**
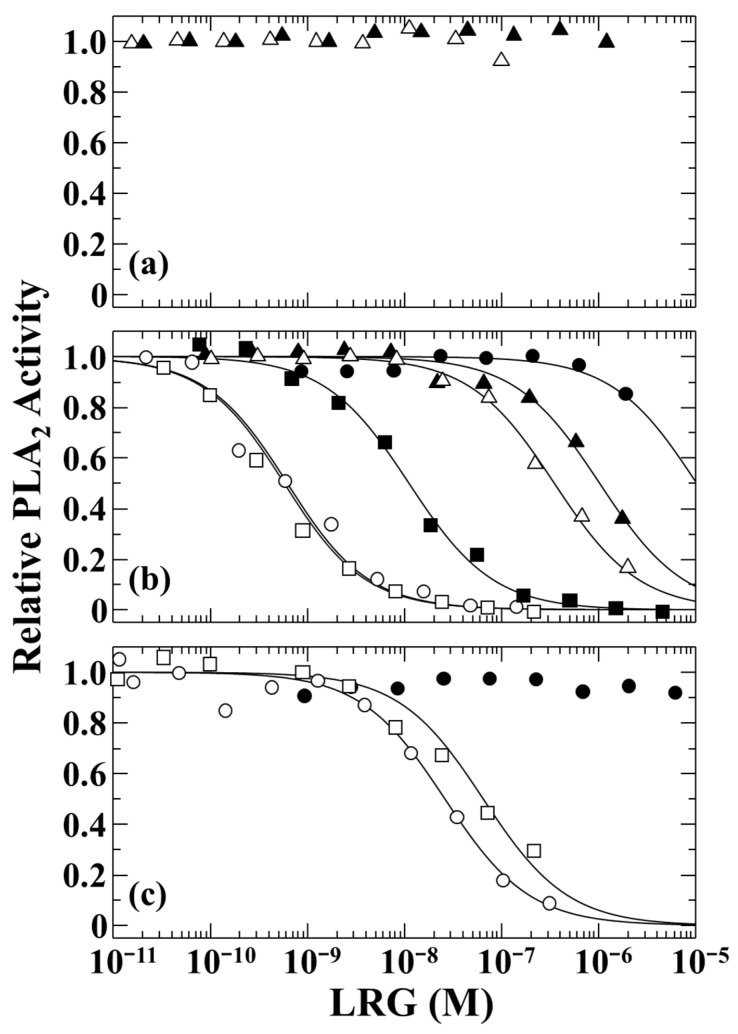
Inhibition of the enzymatic activity of *Laticauda semifasciata* PLA-I (**a**), *Gloydius brevicauda* basic PLA_2_ (**b**), and *Protobothrops flavoviridis* PL-X (**c**) by snake LRGs (PLIβs). The PLA_2_ activity was measured fluorometrically with 10-Pyrene-PC as a substrate, in the presence of various concentrations of LRG. ○, *G. brevicauda* PLIβ; ●, *P. flavoviridis* LRG; □, *Elaphe quadrivirgata* PLIβ (data from ref. [13]); ■, *Elaphe climacophora* PLIβ; △, *Naja kaouthia* LRG; ▲, *L. semifasciata* LRG.

Snake venom PLA_2_s are classified into two groups, I and II. *Elapidae* venom contains group I PLA_2_s, while *Viperidae* venom contains group II PLA_2_s [19]. *G. brevicauda* and *E. quadrivirgata* PLIβ specifically inhibit group II basic PLA_2_s, such as *G. brevicauda* basic PLA_2_ and *P. flavoviridis* PL-X, but do not inhibit group II acidic and neutral PLA_2_ from *G. brevicauda* venom, group I PLA_2_ from *Naja atra* venom, and group III PLA_2_ from honeybee venom [1,13]. *Elapidae* LRGs purified from the sera of *L. semifasciata* and *N. kaouthia* did not inhibit the group I PLA_2_, named PLA-I, which was purified from *L. semifasciata* venom (Figure 2a) but weakly inhibited group II *G. brevicauda* basic PLA_2_ (Figure 2b), with an apparent inhibition constant of 1.03 and 0.35 μM, respectively. Thus, *Elapidae* LRG does not function as the PLA_2_-inhibitory protein, PLIβ. *E. climacophora* PLIβ inhibited *G. brevicauda* basic PLA_2_ but was weaker than *G. brevicauda* and *E. quadrivirgata* PLIβ, with an apparent inhibition constant of 11.1 nM. *P. flavoviridis* LRG hardly inhibited *G. brevicauda* basic PLA_2_ and did not inhibit its own venom PLA_2_, PL-X, although *G. brevicauda* and *E. quadrivirgata* PLIβ inhibited *P. flavoviridis* PL-X, with apparent inhibition constants of 26.1 and 66.0 nM, respectively (Figure 2c). Furthermore, *P. flavoviridis* LRG also did not inhibit *G. brevicauda* acidic PLA_2_ and neutral PLA_2_. This indicated that *P. flavoviridis* LRG does not function as a PLA_2_-inhibitory protein. Since *G. brevicauda* PLIβ purified using a Cyt *c* affinity column in this study showed the same level of PLA_2_ inhibitory activity as the previously reported PLIβ purified by multi-step chromatography, it seems that the PLA_2_ inhibitory activity of *P. flavoviridis* LRG was not lost due to the difference in purification methods.

### 2.3. Surface Plasmon Resonance Analyses of the Interaction of P. flavoviridis LRG with Various Cyt c and PLA_2_

**Figure 3:**
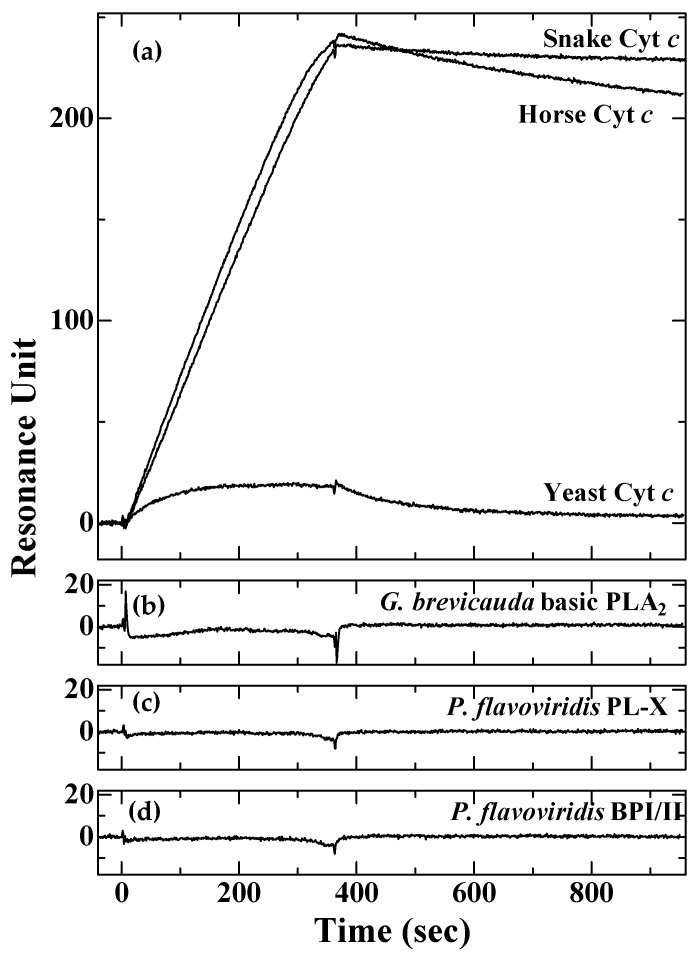
Binding curves for the interactions of Cyt *c* and PLA_2_ with the immobilized *Protobothrops flavoviridis* LRG, measured in real-time using surface plasmon resonance. Sensorgrams for the interaction of immobilized *P. flavoviridis* LRG with (**a**) 5 nM horse heart Cyt *c*, 5 nM *Laticauda semifasciata* Cyt *c*, and 200 nM yeast Cyt *c*, (**b**) 50 nM *Gloydius brevicauda* basic PLA_2_, (**c**) 50 nM *P. flavoviridis* PL-X, and (**d**) 20 nM *P. flavoviridis* BPI/II.

To identify the endogenous ligand of *P. flavoviridis* LRG, we immobilized it on a biosensor chip and measured its interactions with Cyt *c* and PLA_2_. As shown in Figure 3, the sensorgrams indicated that *P. flavoviridis* LRG had a very high affinity for horse and snake Cyt *c*, with a binding manner characterized by fast association and slow dissociation rates. The dissociation constant (*K_d_*) was calculated to be 64.5 pM for horse Cyt c, 14.9 pM for *L. semifasciata* Cyt *c*, and 1.67 μM for yeast Cyt *c*. In contrast, *P. flavoviridis* LRG did not bind to *G. brevicauda* basic PLA_2_ or *P. flavoviridis* PL-X, as expected based on the results of the PLA_2_-inhibitory activity. Because *P. flavoviridis* venom contains myotoxic Lys-49-PLA_2_ homologues, BPI and BPII, we also examined the binding of *P. flavoviridis* LRG to BPI/II, but no binding was detected. The species-specificity of *P. flavoviridis* LRG, which binds more strongly to snake Cyt *c* than to horse Cyt *c*, is consistent with a previous study on *G. brevicauda* PLIβ [17], indicating that the endogenous ligand of LRG is autologous Cyt *c* rather than its venom PLA_2_s.

### 2.4. cDNA Cloning of P. flavoviridis and L. semifasciata LRGs

The full-length cDNA sequence of *L. semifasciata* LRG was determined by means of direct sequencing of the 3′ and 5′ rapid amplification of cDNA ends (RACE) products from sea snake liver cDNA (Supplementary Figure S1). *L. semifasciata* LRG cDNA was 2290 bp (GenBank accession no. LC786336), including 46 bp 5′-untranslated region, 996 bp of the complete coding sequence, and 1145 bp 3′-untranslated region. The predicted open reading frame encodes a protein of 331 amino acids, including a signal peptide consisting of 23 amino acids. Using oligonucleotide primers based on the sequence of *G. brevicauda* PLIβ, we obtained cDNA clones of LRG (LRGcDNA1–4) with four different sequences from reverse transcripts of *P. flavoviridis* liver total RNA (GenBank accession no. LC786337–LC786340). A BLAST search of the nucleotide sequences of these four cDNAs against a draft genome sequence of the habu snake [20] retrieved two LRG genes, which we named *Pf*LRG-A and *Pf*LRG-B. The two LRG genes are located on different chromosomal scaffolds, not tandem duplications, and probably resulted from an ancient duplication event. The nucleotide sequences of *Pf*LRG-A and *Pf*LRG-B differed by 62 bp of the total length of 930 bp, while the amino acid sequences differed by 10 amino acid residues out of the total 310 amino acids (Supplementary Figure S2). LRGcDNA1 and LRGcDNA2 differed from *Pf*LRG-A by one and four bp, respectively, in terms of the nucleotide sequence, resulting in one and two amino acid substitutions, respectively. The nucleotide sequence of LRGcDNA3 was identical to that of *Pf*LRG-B, whereas that of LRGcDNA4 differed from that of *Pf*LRG-B by five bp, resulting in two amino acid substitutions. Thus, LRGcDNA1 and LRGcDNA2 were expected to be variants derived from the *Pf*LRG-A gene, while LRGcDNA3 and LRGcDNA4 were expected to be variants derived from the *Pf*LRG-B gene. Since both the *Pf*LRG-A and *Pf*LRG-B genes are expressed in the liver, we believe that the purified *P. flavoviridis* LRG is a mixture of LRG-A and LRG-B, just as *E. quadrivirgata* PLIβ was a mixture of PLIβ-A and PLIβ-B [13].

### 2.5. Sequence Comparison and Molecular Phylogenic Tree of Snake LRGs

**Figure 4:**
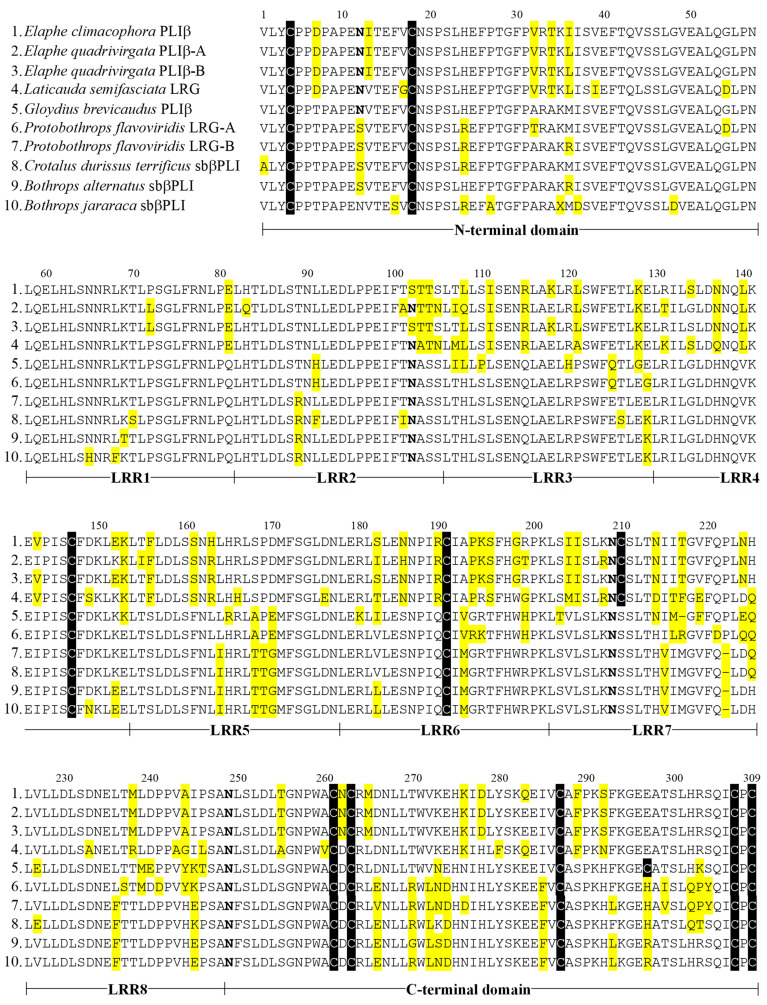
Amino acid sequence alignment of snake LRGs and PLIβs. Cysteine residues are shown with black background, in reversed type. *N*-glycosylation sites are shown in bold type. Residues that differed from those in the other sequences are shown in yellow boxes.

The deduced amino acid sequences of LRGs from *L. semifasciata* and *P. flavoviridis* were compared with those of PLIs from *E. climacophore*, *E. quadrivirgata*, and *G. brevicauda* [11,13,14], and those of sbβPLIs from *C. durissus terrificus*, *B. alternatus*, and *B. jararaca* [16] (Figure 4). The amino acid sequence of *L. semifasciata* LRG reported here is the first in the *Elapidae* family and showed 85–86% homology to those of *Colubridae* LRGs and 72–77% homology to those of *Viperidae* LRGs. Cysteine residues are conserved in all snake LRGs and based on conformational predictions obtained using AlphaFold2 [21], disulfide bonds were predicted to form between Cys3 and Cys18, Cys261 and Cys287, and Cys263 and Cys306. Cys210, which is specific to *Elapidae* and *Colubridae* LRGs, and Cys147 were predicted to occur as free thiol groups, because they are located within the LRR structure. Cys190 and Cys309, which are absent in mammalian LRGs, are likely involved in snake LRG dimer formation.

**Figure 5:**
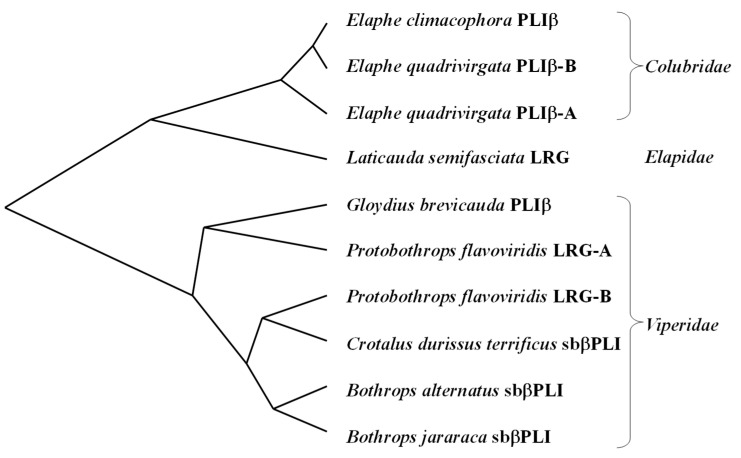
Phylogenetic tree of snake LRGs and PLIβs. The tree was constructed from the nucleotide difference value, based on the sequence alignments shown in Figure 4, according to the neighbor-joining method.

A phylogenetic tree based on the nucleotide sequences encoding mature LRGs was constructed using the neighbor-joining method (Figure 5). Two LRGs, LRG-A and LRG-B, seem to have been generated by gene duplications since the divergence of the two venomous snake families. Both genes are expressed in the liver of *P. flavoviridis*, but only LRG-B genes are expressed as sbβPLIs in the livers of Latin American *Viperidae* snakes. Although sbβPLIs from Latin American *Viperidae* snakes are presumed to inhibit basic PLA_2_ from their own venoms, these have never been purified or characterized; therefore, whether they have PLA_2_-inhibitory activity remains questionable.

## 3. Discussion

By utilizing the strong binding of LRG to Cyt *c*, various snake LRGs can be purified from snake sera in one step, using a Cyt *c* affinity column. Mammalian LRGs are monomers, whereas all snake LRGs purified in the present study were dimers formed by disulfide bonds. Our previous study reported that *G. brevicauda* and *E. quadrivirgata* PLIβ is a trimer based on its molecular weight of 160 kDa from gel-filtration and the results of crosslinking experiments; however, the present results lead us to believe that it is a dimer rather than a trimer, since the molecular weight of LRR proteins upon gel-filtration tends to be larger than the actual molecular weight [22] and the 120 kDa band in the SDS-PAGE of the crosslinking experiment is likely not a trimer. In the previous multi-step purification procedure, only 0.1 mg of LRG (PLIβ) was purified per 10 mL of *E. climacophora* serum [12]; however, in the current one-step procedure using the Cyt *c* affinity column, the yield was 0.4 mg. Purification by means of one-step chromatography rather than multi-step chromatography is expected to significantly increase the recovery of LRG, thereby allowing for estimation of the amount of LRG in the serum. Table 1 shows the recovery of LRG purified from 10 mL snake sera in the present study. Although 0.16 mg of *E. quadrivirgata* PLIβ could be purified from 2 mL of serum using a multi-step purification procedure [13], it is expected that several milligrams of PLIβ can be obtained from 10 mL of serum, if purified using the present one-step purification procedure. Unlike *E. climacophora*, *E. quadrivirgata* has an ophiophagous habit and often feeds on the Japanese mamushi *Gloydius blomhoffii* [23]. Therefore, *E. quadrivirgata* PLIβ probably functions as a defense against envenomation. Thus, the higher amounts of *G. brevicauda* and *E. quadrivirgata* PLIβ in the serum, compared to the amounts of LRG/PLIβ in the serum of other snakes, are probably due to the protective function of neutralizing basic PLA_2_ in mamushi venom, in addition to LRG’s original function of binding Cyt *c*. It has been shown that intramuscular injection of *G. brevicauda* venom into *G. brevicauda* enhances the gene expression of PLIα and PLIβ in the liver [24].

Although the amino acid sequences of *E. quadrivirgata* PLIβ-B and *E. climacophora* PLIβ differ by only four residues (positions 36, 72, 163, and 283), the apparent inhibition constant of *E. climacophora* PLIβ for *G. brevicauda* basic PLA_2_ was approximately 20-fold greater than that for *E. quadrivirgata* PLIβ. This difference in the PLA_2_-inhibitory activity may reflect the difference in the inhibitory activity between *E. quadrivirgata* PLIβ-A and PLIβ-B, because *E. quadrivirgata* PLIβ was a mixture of PLIβ-A and PLIβ-B [13].

Despite its high homology (87.6%) with *G. brevicauda* PLIβ, *P. flavoviridis* LRG neither bound to nor inhibited its basic PLA_2_, PL-X, BPI/II, or *G. brevicauda* basic PLA_2_. A comparison of the amino acid sequences of *G. brevicauda* PLIβ and *P. flavoviridis* LRG-A revealed the following amino acid differences: 7.0% in the N-terminal region, 0% in LRR1, 0% in LRR2, 25.0% in LRR3, 4.2% in LRR4, 4.2% in LRR5, 16.7% in LRR6, 29.2% in LRR7, 20.8% in LRR8, and 16.7% in the C-terminal region. This may indicate that the C-terminal region of *G. brevicauda* PLIβ (especially LRR7, LRR8, and the C-terminal domain) is involved in the binding to *G. brevicauda* basic PLA_2_. There were 13 amino acid residues (positions 12, 24, 153, 214, 266, 270, 272, 274, 285, 297, 299, 302, 303, 304) that were substituted in *P. flavoviridis* LRG, even though they are commonly conserved in *G. brevicauda* PLIβ and *E.quadrivirgata* PLIβ. A total of 9 of these 13 unique amino acid substitutions are located in the C-terminal domain, also indicating that the C-terminal region is involved in the inhibition of *G. brevicauda* PLA_2_. In contrast, Fortes-Diaz et al. [16] predicted that the negatively charged area located at the N-terminal region and LRR1–5 on the concave surface of the *C. durissus terrificus* sbβPLI in silico model is the binding region for basic PLA_2_s, although the binding activity of *C. durissus terrificus* sbβPLI to basic PLA_2_ has not been confirmed. Because Cyt *c* and basic PLA_2_ are both basic soluble proteins with nearly the same molecular weight, the Cyt *c*-binding protein, LRG, probably has potential binding activity to basic PLA_2_, which could explain why *N. kaouthia*, *L. semifasciata*, and *P. flavoviridis* LRGs inhibited *G. brevicauda* basic PLA_2_ with apparent inhibition constants in the micromolar levels. Thus, we concluded that snake LRGs have the potential to inhibit basic PLA_2_; however, whether they actually function as PLIβ, a PLA_2_-inhibitory protein, varies from snake to snake. For *G. brevicauda* and *E. quadrivirgata*, the evolutionary process may have altered their LRGs to increase their binding to *G. brevicauda* basic PLA_2_ and used the LRG as a PLIβ, one of the defense mechanisms against venom.

## 4. Material and Methods

### 4.1. Materials

Bloods from Erabu sea krait (*L. semifasciata*), habu (*P. flavoviridis*), monocled cobra (*N. kaouthia*), Chinese mamushi (*G. brevicauda*), and Japanese rat snake (*E. climacophora*) were collected at the Japan Snake Institute (Gunma, Japan). *G. brevicauda* basic PLA_2_ was purified from *G. brevicauda* venom, as described previously [6]. *L. semifasciata* PLA_2_, named PLA-I, was purified as described previously [25]. *P. flavoviridis* PL-X and BPI/II were purified by means of reverse-phase high-performance liquid chromatography from the fractions eluted with buffers containing 0.4 and 0.5 M NaCl, using CM-cellulose chromatography, during the purification process of *P. flavoviridis* PL-X, as described previously [6]. *P. flavoviridis* BPI and BPII, which differ by only one amino acid residue [26], could not be separated by means of reverse-phase high-performance liquid chromatography, and the fractions obtained were designated as BPI/II, because they were considered to contain both BPI and BPII. Horse heart Cyt *c* was purchased from Wako Pure Chemical Industries. Ltd. (Osaka, Japan) and yeast Cyt *c* was procured from Sigma-Aldrich (St. Louis, MO, USA). *L. semifasciata* Cyt *c* was purified from frozen hearts, as described previously [17]. All other reagents and chemicals used were of the highest quality available.

### 4.2. Purification of LRGs from Various Snake Sera

LRGs were purified from various snake serum samples using a Cyt *c* affinity column. Horse heart Cyt *c* was coupled to a HiTrap NHS-activated HP column (GE Healthcare, Buckinghamshire, UK) to produce a Cyt *c* affinity column, as described previously [17]. Sera from various snakes were loaded into the Cyt *c* affinity column, equilibrated with 20 mM sodium phosphate buffer (pH 7.4) containing 0.15 M NaCl, and washed with the same buffer. LRG was eluted with 0.1 M sodium acetate buffer (pH 4.0) containing 0.5 M NaCl. SDS-PAGE was analyzed under reducing (5% β-mercaptoethanol) and non-reducing conditions, using a 10% polyacrylamide gel. Protein bands were stained with Quick CBB (Wako Pure Chemical Industries). The N-terminal sequences of the LRGs were determined using a protein sequencer (491HT; Applied Biosystems, Foster City, CA, USA).

### 4.3. Inhibition of PLA_2_ Enzymatic Activity

PLA_2_ activity was measured fluorometrically using 1-palmitoyl-2-(10-pyrenyldecanoyl)-*sn*-glycero-3-phosphoryl-choline (10-Pyrene-PC; Cayman Chemical, Ann Arbor, MI, USA) as a substrate, as described previously [6] in the presence of various concentrations of LRGs. The apparent inhibition constant (*K*_i_^app^) was determined by means of non-linear least-squares analysis of relative PLA_2_ activity.

### 4.4. Binding Analysis Using Surface Plasmon Resonance

The BIAcore^®^ X System (GE Healthcare) was utilized to study the interaction of *P. flavoviridis* LRG with Cyt *c* and PLA_2_. *P. flavoviridis* LRG was coupled to a CM5 sensor chip using an amine coupling kit according to the manufacturer’s instructions. Binding experiments were performed at 25 °C, using HEPES running buffer (50 mM HEPES buffer containing 0.05% Tween 20, pH 7.5, with NaCl added to obtain an ionic strength of 0.2). Cyt *c* or PLA_2_ samples at different concentrations were perfused over the sensor chip surface to obtain real-time binding data at a flow rate of 10 μL/min. The samples are replaced by running buffer in the dissociation phase. The sensor chip was regenerated by injection of a 10 mM sodium acetate buffer (pH 4.0) containing 0.5 M NaCl. All experiments used repetitive cycles of the same injection and regeneration protocol. The association and dissociation curves were analyzed with BIAevaluation 3.0, using the 1:1 Langmuir binding model with a drifting baseline (global fitting). The apparent *K_d_* values were calculated from the association rate and dissociation constants.

### 4.5. cDNA Cloning and Sequence Analysis

Total RNA was isolated from *L. semifasciata* or *P. flavoviridis* liver using a RNeasy Mini Kit (Qiagen, Valencia, CA, USA), according to the manufacturer’s instructions. A SMARTer™ RACE cDNA Amplification Kit (Clontech Laboratories, Palo Alto, CA, USA) was used to clone the 3′- and 5′-ends of *L. semifasciata* LRG cDNA. The first-strand 3′- and 5′-RACE-ready cDNA samples were prepared according to the manufacturer’s protocol and used as templates for 3′- and 5′-RACE, respectively. The first and nested PCR amplification of *L. semifasciata* LRG cDNA 3′- and 5′-ends was carried out with the Advantage™ 2 PCR Kit (Clontech Laboratories) and two primers, *Ls*β1 (5′-CCCTTCCGAGTGGCCTCTTCCGTA-3′) and *Ls*β2 (5′-CAGGTTAGAAGATTGTCCATGCGGCAGT-3′), corresponding to sequences 323–346 and 926–899, respectively, of *E. climacophora* PLIβ cDNA (GenBank accession no. AB462511). The PCR products were subjected to electrophoresis on 1.4% agarose gels and extracted using an Agarose Gel Extraction Kit (Roche Diagnostics, Mannheim, Germany). Total RNA from *P. flavoviridis* liver was reverse transcribed to cDNA using the PrimeScript RT Reagent Kit (Takara, Kusatsu, Japan). The cDNA fragments of *P. flavoviridis* LRG were amplified by means of PCR, using the following primer pair: *Pf*β1 (5′-GGCAGGGTGTCCAGCGTCCTTTACTGCCCACCC-3′) and *Pf*β2 (5′-GCCCTCTAGACTCGAGTTAGCAGGGACAAATTTGGT-3′), designed based on the cDNA sequence of *G. brevicauda* PLIβ (GenBank accession no. AB007198). *P. flavoviridis* LRG cDNA fragments with 15 bp flanking sequences homologous to the vector ends were integrated into the plasmid pcDNA3.1, using the In-Fusion^®^ HD Cloning Kit (Clontech Laboratories), and transformed into *Escherichia coli* DH5α Competent Cells (Takara). The PCR products and plasmids were sequenced using the DYEnamic ET Terminator Cycle Sequencing Premix Kit (GE Healthcare) on ABI 310 and 3500 Genetic Analyzers (Applied Biosystems). The cDNA sequences determined in the present study were deposited in the DDBJ/EMBL/GenBank nucleotide sequence databases under accession nos. LC786336 (*L. semifasciata* LRG) and LC786337–LC786340 (cDNA1–4 of *P. flavoviridis* LRG). Analysis of the DNA sequence data, alignment of the amino acid sequence, and construction of a phylogenetic tree were performed using GENETYX ver. 6 (Genetyx, Tokyo, Japan).

### 4.6. Protein Structure Prediction

Protein structures and complexes were predicted using AlphaFold2 [21] and AlphaFold2-multimer [27]. Sequence alignments and templates were generated using MMseqs2 and HHsearch. Both multiple sequence alignment and AlphaFold2 predictions were performed using ColabFold [28].

## Figures and Tables

**Table 1:** The LRG amounts recovered from 10 mL sera of various snakes, by means of a one-step purification procedure using a Cyt *c* affinity column.

Snakes	LRG Amount (mg/10 mL Serum)
*Laticauda semifasciata*	0.67
*Naja kaouthia*	0.48
*Elaphe climacophore*	0.40
*Gloydius brevicauda*	4.30
*Protobothrops flavoviridis*	0.17

## Data Availability

Data are contained within the article and Supplementary Materials.

## Supplementary Materials

The following supporting information can be downloaded at: https://www.mdpi.com/article/10.3390/toxins16030126/s1; Figure S1: Nucleotide and deduced amino acid sequences of Laticauda semifasciata LRG; Figure S2: Comparison of nucleotide sequences of *Protobothrops flavoviridis* LRGs.
