# Supplementary material for: One-Step Affinity Purification of Leucine-Rich α2-Glycoproteins from Snake Sera and Characterization of Their Phospholipase A2-Inhibitory Activities as β-Type Phospholipase A2 Inhibitors
Source: Toxins (Basel). 2024 Mar 1;16(3):126. doi: 10.3390/toxins16030126 (PMC10975490; doi:10.3390/toxins16030126)
Supplement: Supplementary file 1 [file toxins-16-00126-s001.zip › toxins-2841930-supplementary.pdf]

Supplementary Materials: One-Step Affinity Purification of Leucine-Rich α2-Glycoproteins from Snake Sera and Characterization of Their Phospholipase A2-Inhibitory Activities as β-Type Phospholipase A2 Inhibitors

Ryoichi Shirai, Kana Shibata, Shinobu Fujii, Rikiro Fukunaga and Seiji Inoue

1 AGTGTCCAAAAGGCTGGCAGGAGTGAGGTTTGGACAGAGTTCCAGGATGAAGTCT 55

(-23) M K S (-21)

56 TCGGTTCTAGCACTTCTGATCATCTGCCTTGTGATGTCCTTCAACAGCTACACCCAGCAGGTTCTT 121

(-20) S V L A L L I I C L V M S F N S Y T Q Q V L (2)

122 TATTGCCCACCCGATCCTGCTCCAGAAAATATCACTGAGTTTGGCTGCAACTCCCCATCTCTCCAT 187

(3) Y C P P D P A P E **N** I T E F G C N S P S L H (24)

188 GAATTTCCCACTGGCTTCCCTGTACGAACCAAACTTATCTCCATTGAGTTCACCCAGCTCTCCAGC 253

(25) E F P T G F P V R T K L I S I E F T Q L S S (46)

254 CTTGGCGTGGAAGCCCTCCAAGATCTTCCAAACCTCCAGGAACTTCACCTCTCCAACAACAGGCTG 319

(47) L G V E A L Q D L P N L Q E L H L S N N R L (68)

320 AAGACCCTTCCCAGTGGCCTCTTCCGTAACCTCCCAGAATTGCACACCCTGGATCTGTCCACCAAT 385

(69) K T L P S G L F R N L P E L H T L D L S T N (90)

386 CTCCTGGAAGATCTACCCCCAGAGATCTTCACCAACGCAACCAACCTGATGCTTTTATCCATCAGT 451

(91) L L E D L P P E I F T **N** A T N L M L L S I S (112)

452 GAAAACCGACTGGCTGAGCTGCGGGCATCCTGGTTCGAAACCCTGAAGGAACTTAAGATCCTGAGC 517

(113) E N R L A E L R A S W F E T L K E L K I L S (134)

518 CTTGACCAAAACCAGCTGAAGGAGGTCCCCATTTCTTGTTTCTCCAAGCTGAAGAAGTTGACCTTT 583

(135) L D Q N Q L K E V P I S C F S K L K K L T F (156)

584 CTAGATCTCTCCTCCAATCGCCTCCATCATCTCTCTCCAGACATGTTCAGCGGCCTAGAAAATTTG 649

(157) L D L S S N R L H H L S P D M F S G L E N L (178)

650 GAGAGGTTGACCTTGGAAAATAACCCAATCCGATGCATTGCCCCGAGATCCTTCCATTGGGGACCC 715

(179) E R L T L E N N P I R C I A P R S F H W G P (200)

716 AAGCTGAGCATGATCTCCCTGAGGAACTGCAGCCTGACCGATATCACCTTTGGGGAATTTCAGCCG 781

(201) K L S M I S L R **N** C S L T D I T F G E F Q P (222)

782 TTGGACCAACTGGTGCTGCTGGATCTCTCCGCCAATGAGCTCACCAGGCTGGATCCTCCAGCCGGC 847

(223) L D Q L V L L D L S A N E L T R L D P P A G (244)

848 ATCCTGTCTGCCAATCTCAGCCTGGATCTTGCAGGAAATCCTTGGGTGTGTGACTGCCGCCTGGAC 913

(245) I L S A **N** L S L D L A G N P W V C D C R L D (266)

914 AATCTTCTAACCTGGGTCAAGGAGCACAAGATCCATTTATTTTCCAAGCAGGAAATTGTCTGTGCT 979

(267) N L L T W V K E H K I H L F S K Q E I V C A (288)

980 TTCCCCAAGAATTTCAAAGGCGAAGAGGCAACCTCACTTCATCGATCCCAAATTTGTCCCTGCTAA 1045

(289) F P K N F K G E E A T S L H R S Q I C P C (309)

1046 ACCTTTAAATAGATCCTTTTCTTTCCACCCTGCTTCGCGAACAGCGAGATTTCCCAGTTTGGAGAC 1111

1112 AATTTCCAGTTGAATTGGACTTCAACTCCCAGAAATCTCAGCAACAGCCATGCTGCCTGGGGAATT 1177

1178 CTGGGAGTTGGAGTCCACATAGCTGGATTTAAAGAGAAGAGACAGAACAGGATTGGGAAAAAAAAT 1243

1244 ACGGAAATACTGATTTTAGGGAAGAAATAGCTGCTCAGGAAGAGATGCTCAAAAAAATCAACAATA 1309

1310 TAGGTAGTCCTTCACTTACAACCGTTCAGTTAGTGACTTTTTGAAGTATTAAGAGCCCTGAAGAAA 1375

1376 GTGACCGATGAGTGGATCAGAATCAGAATAGAGCTGCAAGGGACCTTGGAGGTCTTCTAGTCCAAC 1441

1442 CCCCTGCTCAAGCAGGAGACCCTCTGCCATTTCAGACAAGCAACTGTCAGTCTCTTCTTGAAAACT 1507

1508 GATGGAGCACCACAACTTCTGGTGGCAAGCTGTTCCACTGATTAATTGTTCCCTGTCAGGAAGTTT 1573

1574 CTCCTTAGTTCTAGGTTGCTTCCCTCCTTGATTAATTTCCATCCTTTGCTTCTTGTCCTGCCCTCA 1639

1640 GGTGCTTTGAAGAATAGCTTGACTCCCTCTTCTTTGTGGCAGCCCCTCAAATATTGGAAGACTGAT 1705

1706 ATCATGTCACCCTTAGTGCTTCTCAGAGCTGACTGTTTTGCTGTTTCTATTATTATTAGTGTAGGG 1771

1772 TGCTGTTACATTAGCCAGGAGTCTGCAATGCTACTGAAGCGTAGTACTTTGCCTGCAAATCTACTA 1837

1838 GCTCTGTTATGGTACATGCTCACGCACATCCCAAGGTGCACCACAGGAGAGCGAGCTAGCTTTGCA 1903

1904 ACAGAAAGAAAGACAAGCAGCTTTTCCTGCAGCATTAGCCAAGCCCTTTCCCCTCCCTCCACCGTG 1969

1970 ACCACCTCGCAAGCGGGCATCGCAAATGGGAAGGTTCAAAGCGGACAGAAATTTGACTGCCTGCAC 2035

2036 AGAGCACCAAGTCGTCTCCCCCTCAAAGTGGTACCTATTTGTCTACAGGCGGTATATATGATTTTG 2101

2102 AGCTGCTAGGTGGGCAGAAGCTGAGGCAAGTTCGGGAGCTCATCCTGCTACGCAGTGCTAGGGCTC 2167

2168 GAACCACTGGAGCAGAGACCAACTCCAGCATCATTAAGCCACTGAGCCAATGTACTTCTCTGTTTC 2233

2234 GCTGTTGAATAAACCTTATTTCCACTTCAAAAAAAAAAAAAAAAAAAAAAAAAAAAA 2290

Supplementary Figure S1. The nucleotide and deduced amino acid sequences of Laticauda semifasciata LRG. The N-terminal sequence elucidated by sequencing the purified L. semifasciata LRG is underlined. Potential N-glycosylation sites are indicated in bold.

```
PfLRG-A   1 GTCCTTTACTGCCCACCCACCCCTGCTCCAGAAAGTGTCACCGAGTTCGTCTGCAACTCTCCATCTCTACGTGAATTTCCCACCG 85
cDNA1     1 ..................................................................................... 85
cDNA2     1 ..................................................................................... 85
PfLRG-B   1 ..................................................................................... 85
cDNA3     1 ..................................................................................... 85
cDNA4     1 ..........................C...A....C................................................. 85

PfLRG-A  86 GCTTCCCCACACGAGCCAAGATGATCTCCGTTGAGTTCACCCAAGTCTCCAGCCTTGGCGTGGAGGCCCTCCAAGATCTTCCAAA 170
cDNA1    86 ..................................................................................... 170
cDNA2    86 ..................................................................................... 170
PfLRG-B  86 .......TG............G.....................G..T............................G......C.. 170
cDNA3    86 .......TG............G.....................G..T............................G......C.. 170
cDNA4    86 .....................G.....................G..T............................G......C.. 170

PfLRG-A 171 CCTCCAGGAACTTCACCTCTCCAACAACAGGTTAAAAACTCTTCCAAGTGGCCTCTTCCGTAACCTCCCACAACTGCACACCTTG 255
cDNA1   171 ..................................................................................... 255
cDNA2   171 ..................................................................................... 255
PfLRG-B 171 ..................................................................................... 255
cDNA3   171 ..................................................................................... 255
cDNA4   171 ..................................................................................... 255

PfLRG-A 256 GATCTCTCCACAAATCATCTAGAAGATCTACCTCCAGAGATCTTTACAAATGCAAGTAGCCTAACTCATTTATCCCTCAGTGAAA 340
cDNA1   256 ..................................................................................... 340
cDNA2   256 ..................................................................................... 340
PfLRG-B 256 ..........G.....TC..........................C........................................ 340
cDNA3   256 ..........G.....TC..........................C........................................ 340
cDNA4   256 ..........G.....TC..........................C........................................ 340

PfLRG-A 341 ATCAACTAGCTGAACTGCGCCCATCCTGGTTCCAAACTCTTGAAGGACTCAGGATCCTAGGCCTTGATCACAATCAGGTGAAGGA 425
cDNA1   341 ..................................................................................... 425
cDNA2   341 ..................................................................................... 425
PfLRG-B 341 .......G.......................TG....C..G..G.A....................................... 425
cDNA3   341 .......G.......................TG....C..G..G.A....................................... 425
cDNA4   341 .......G.......................TG....C..G..G.A....................................... 425

PfLRG-A 426 GATCCCAATTTCTTGTTTTGATAAGCTGAAGGAGTTGACATCTCTAGATCTCTCATTCAACCTCCTCCATCGCCTCGCTCCAGAG 510
cDNA1   426 ..................................................................................... 510
cDNA2   426 ..................................................................................... 510
PfLRG-B 426 ............................................................T...A...........A..A...G. 510
cDNA3   426 ............................................................T...A...........A..A...G. 510
cDNA4   426 ............................................................T...A...........A..A...G. 510

PfLRG-A 511 ATGTTCAGTGGCTTAGATAATTTGGAGAGGTTAGTGCTGGAAAGCAACCCCATCCAGTGCATTGTCAGGAAGACCTTCCATTGGC 595
cDNA1   511 ..................................................................................... 595
cDNA2   511 .................................C.T................................................. 595
PfLRG-B 511 .................C.................T...........T..A........T...A.GG...G.............. 595
cDNA3   511 .................C.................T...........T..A........T...A.GG...G.............. 595
cDNA4   511 .................C.................T...........T..A........T...A.GG...G.............. 595

PfLRG-A 596 ATCCCAAGCTGAGTGTGCTGTCCCTGAAGAACAGCAGCCTGACCCACATCTTAAGGGGAGTCTTTGACCCATTGCAACAACTGGT 680
cDNA1   596 ..................................................................................... 680
cDNA2   596 ......................................................C.............................. 680
PfLRG-B 596 G..................A..........................TG..A...T...G......C.G---...G.C........ 677
cDNA3   596 G..................A..........................TG..A...T...G......C.G---...G.C........ 677
cDNA4   596 G..................A..........................TG..A...T...G......C.G---...G.C........ 677

PfLRG-A 681 GCTGCTGGACCTCTCTGACAATGAGCTCAGCACAATGGATGATCCAGTCTACAAGCCGTCTGCCAATCTCAGTCTTGATCTTTCA 765
cDNA1   681 ..................................................................................... 765
cDNA2   681 ...............C..................................................................... 765
PfLRG-B 678 .........................T...C....C.....CC...G...C..G....A.........T................. 762
cDNA3   678 .........................T...C....C.....CC...G...C..G....A.........T................. 762
cDNA4   678 .........................T...C....C.....CC...G...C..G....A.........T................. 762

PfLRG-A 766 GGAAACCCTTGGGCGTGTGACTGCCGCCTGGAGAATCTTCTAAGATGGCTCAATGATCACAACATCCATTTATATTCTAAGGAGG 850
cDNA1   766 .................................................................................A... 850
cDNA2   766 ..................................................................................... 850
PfLRG-B 763 ..........................G....T............................G................C....... 847
cDNA3   763 ..........................G....T............................G................C....... 847
cDNA4   763 ..........................G....T............................G................C....... 847

PfLRG-A 851 AATTTGTCTGTGCTTCCCCCAAGCATTTCAAGGGTGAACATGCAATTTCACTTCAACCATACCAAATTTGTCCCTGCTAA      930
cDNA1   851 ................................................................................      930
cDNA2   851 ................................................................................      930
PfLRG-B 848 ............................G...............G...................................      927
cDNA3   848 ............................G...............G...................................      927
cDNA4   848 ............................G...............G...................................      927
```

Supplementary Figure S2. Comparison of the nucleotide sequences of Protobothrops flavoviridis LRGs.

PfLRG-A and PfLRG-B were located on habu1_scaffold2243:7438-6509 and habu1_scaffold11684:3373-2447, respectively, in the draft genome sequence of the habu snake P. flavoviridis. Four cDNA clones 1–4 were obtained from the total liver RNA. Sequence identities have been represented by dots.
